# Beyond a digital habit: Socio-ecological perspectives on social media disorder among Indonesian college students

**DOI:** 10.3934/publichealth.2025057

**Published:** 2025-11-27

**Authors:** Ulfi Hida Zainita, Evi Martha, Tiara Amelia, Dewi Safitri

**Affiliations:** Department of Health Education and Behavioral Sciences, Faculty of Public Health, Universitas Indonesia, Building D 1st Floor (16424), Depok, West Java, Indonesia

**Keywords:** social media disorder, social media addiction, college student, young adult, mental health

## Abstract

**Background:**

In Indonesia, with a total population of 278.7 million, it was recorded that almost half the population were social media users (49.9%) in 2024. Studies from various sources report that passive and excessive use of social media, especially in the student age category, is a trigger for Social Media Disorder (SMD). However, research examining the socio-cultural factors of SMD is lacking, especially in the Indonesian context. Thus, in this study, we aimed to explore the phenomenon of SMD to reveal the social and cultural aspects among college students.

**Methods:**

We conducted a qualitative case study, and data were obtained using semi-structured online interviews conducted in April-June 2025. A total of 12 informants (Code: I 1–12) aged 18–22 years scored 5–9 using the SMD scale from 12 faculties at the Universitas Indonesia. The data was collected through semi-structured online interviews. The data were analyzed using reflexive thematic analysis.

**Results:**

All participants had accounts on WhatsApp, LINE, Instagram, TikTok, X (formerly Twitter), and YouTube. Nine common subthemes were identified across participants: (1) ‘Duration of social media use’, (2) ‘The second accounts of Instagram’, (3) ‘Each social media has different motivation uses’, (4) ‘Passive activity on social media’, (5) ‘Importance of peer groups’, (6) ‘Lack of openness toward parents’, (7) ‘Collective efficacy gives courage to speak up’, (8) ‘Social etiquette is a must’, and (9) ‘The shifting of cultural value during social media use’.

**Conclusion:**

Social media use among students was deeply embedded in their academic routines, emotional coping strategies, and social navigation. Ultimately, addressing SMD requires a shift from simplistic models of addiction toward a more nuanced understanding that integrates cultural context, peer dynamics, and emotional well-being.

## 1. Introduction

Social media is one of the technologies that changes the trend of human communication. Social media is defined as an online platform where users can communicate without being limited by space and time [1]. Users have various purposes for using social media, such as socializing, spending free time, seeking entertainment, convenience, sharing news, sharing photos or videos, playing games, making posts, and searching for information [2],[3].

The number of social media users in the world increased from 4.2 billion in 2021 to 5 billion users in 2024 [4]. Moreover, in Indonesia, with a total population of 278.7 million, it was recorded that almost half the population, about 139 million, were social media users (49.9%) in 2024 [4]. A survey of internet users conducted by the Indonesian Internet Service Providers Association reported that the highest age group of internet users was Generation Z (born 1997–2012), with a usage of 87.02%, while the Millennial Generation (born 1996–1981) was 79.5%, and Generation X (born 1965–1980) was 60.52% [5].

A report by APJII (2024) shows that social media usage has increased among college students in Indonesia from 82.46% in 2021 to 87.02% in 2024, but research examining the socio-cultural factors of Social Media Disorder (SMD) is lacking, especially in the Indonesian context. In this study, we seek to identify this gap by examining these factors among college students. Understanding the factors that contribute to SMD in this context is critical, especially as college students navigate the complexities of modern digital life within a traditional socio-cultural framework in Indonesia.

Studies from various sources report that passive and excessive use of social media, especially in the student age category, is a trigger for SMD [6]–[8]. SMD is a mental health disorder that is not desired and can affect other mental health disorders, such as depression, anxiety, impaired concentration during lectures, Fear of Missing Out (FOMO), decreased quality of life, and others. Although there have been many studies related to SMD, there are few studies that explore socio-cultural factors related to SMD.

A study by Van Den Eijnden, Lemmens, and Valkenburg (2016) explains that the definition of SMD is if at least five of the nine disorder criteria are found in an individual in the past year [9]. These nine criteria are; preoccupation, tolerance, withdrawal, persistence, displacement, problem, deception, escape, and conflict. These criteria refer to the criteria for Internet Gaming Disorder (IGD), because IGD is part of the internet addiction construct that has been officially recognized by the Diagnostic and Statistical Manual of Mental Disorders Fifth Edition (DSM-5). The number of question items on the SMD Scale is 9 items, with yes and no answer options. If you answer yes ≥5, then it is categorized as SMD [9].

SMD is a relatively new issue, so there is little literature examining the factors that influence SMD. Researchers have found several factors that influence SMD in students, namely age, gender, ethnicity, duration of social media use, number of social media accounts, having activities such as work and organizations, duration of social media use, and the number of accounts owned by individuals [6],[7],[10],[11].

A study related to SMD was conducted at the Universitas Indonesia in 2021, but it did not examine socio-cultural factors [6]. Sun and Zhang stated that studies related to SMD mostly focus on personal-level factors, such as individual psychology, dispositional differences, and cognitive factors [12]. Moreover, there are many other broader socio-cultural factors that have a significant contribution to SMD that have not been studied in depth, such as sociodemographics, peer influence, communication style, collective efficacy, cultural values, religious values, social norms, and others [13]–[16].

Socio-cultural factors are defined as environmental conditions that play a role in a person's behavior, well-being, health, and adaptive abilities [17]. The socio-ecological model (SEM) explains that health is influenced by the interaction between three scopes, namely individual characteristics, communities, and the environment, which includes physical, socio-cultural, and political components [13],[14]. SEM is a comprehensive framework for understanding the multifaceted nature of addictive behavior by examining the interaction between individual, interpersonal, community, and environmental factors, including socio-cultural and societal interactions [18]. Socio-cultural factors are one of the strengths of SEM because they cover four scopes, namely individuals, social network, community, and societal factors, which then consist of several socio-cultural variables such as sociodemographics, peer influence, communication style, family relationships, relationships with friends, collective efficacy, cultural values, religious values, social norms, gender norms, and political policies [13]–[15].

Socio-cultural factors are one of the strengths of SEM in identifying SMD in students because they cover four scopes, namely the individual scope (duration of social media use, number of accounts, motivation for using social media, and social media use activities), social network scope (peer influence and family relationships), community scope (collective efficacy), and societal scope (social norms and cultural values) [14],[15],[19]. Based on the description above, a qualitative study needs to be conducted to prevent SMD and other negative impacts of social media among students. Here, we aim to explore the phenomenon SMD to reveal the social and cultural aspects among students who have SMD. This qualitative study is needed as basic data for policy making related to social media in the future by considering the social and cultural factors of students, especially UI undergraduate students.

## 2. Materials and methods

### 2.1. Research type

A qualitative case study was conducted to answer the following research questions: 1) How socio-cultural aspects shape the social media use behavior among college students who have SMD; and 2) how the socio-ecological model reveals the interplay within individual scope, social network scope, community scope, and societal scope among college students who have SMD. The data was obtained through online in-depth interviews with a semi-structured guide and was analyzed thematically. This study was conducted in April–June 2025.

### 2.2. Population and informants

We used a qualitative case study method on 12 student informants (Code: I 1–12) aged 18–22 years who experienced SMD with a score of >5 (obtained through secondary data from other research in March 2025) from 12 faculty at the Universitas Indonesia using purposive sampling to ensure diversity in socio-cultural backgrounds. We selected 12 informants because we found that the data obtained was adequate and saturated. The purposeful sampling of participants was based on the inclusion criteria of the study and the availability of those who were willing to participate.

### 2.3. Research location

The study was conducted in Universitas Indonesia, a culturally diverse university with a mix of Indonesian youth, which provided a unique context for understanding how the socio-cultural aspects shape social media use behavior and exploring the socio-ecological model that interplays within individual scope, social network scope, community scope, and societal scope among college students who had SMD.

### 2.4. Instrumentation

A semi-structured IDI guide (Supplementary 1) was developed to explore the participants' experiences, focusing on the interactions of socio socio-ecological model and social media disorder experience.

### 2.5. Data collection procedures

Stage 1. Student screening with the SMD scale. Data was obtained from research conducted in March 2025 by Zainita (2025) [20].

a) A sample of UI undergraduate students in 12 faculties using convenience sampling techniques filled out the social media disorder Scale by Van Eijden (2016), which was translated into Indonesian and was tested for validity and reliability by Dewi & Lestari (2020).

b) Contacting students who had SMD scores of 5–9 and were willing to conduct in-depth interviews.

Stage 2. Qualitative Data Collection

a) Conduct in-depth interviews with students who experienced SMD via the online Zoom platform, lasting approximately 60–90 minutes each.

b) Semi-structured interview guide (Supplementary 1) with open questions, which enabled flexibility by giving the opportunity to the participants to freely answer and respond.

c) The interviews were recorded using recording features from the Zoom meeting application.

### 2.6. Data analysis

The analysis was reported as a series of case studies with comparative data analyzed using reflexive thematic analysis, in which participants' responses were interpreted to reflect their lived experience [21]. We took this approach to give voice to participants' individual stories, given the paucity of published research examining the experiences of college students experiencing SMD. Also, utilizing reflexive thematic analysis then enabled us to comprehensively explore participants' shared experiences. Braun & Clarke (2021) outlined that reflexive thematic analysis follows a six-step process:

a) According to their guide, we conducted data familiarization using audio recordings that were transcribed verbatim into Microsoft Word.

b) The second step involves importing verbatim into NVivo version 12 to aid in organizing, coding, and retrieving the data.

c) Interesting aspects are identified and data relevant to each code are gathered, sorted, and then collating similar coded data into subthemes.

d) At the next step, subthemes are checked for forming a coherence pattern and their relation to the extracts. The overarching themes also formed.

e) Then researchers define and name the subthemes and overarching themes in a way that gives the reader right away the idea of what the theme is about.

f) The final step includes presentation of nine subthemes, four overarching themes, and representative quotes in a report. The researchers then met to cross-check the identified patterns and compare the results. Discrepancies that occurred were discussed and resolved by all authors.

### 2.7. Ethics approval of research

This study has been given ethical approval from the Health Research Ethics Board of Universitas Muhammadiyah Prof. Dr HAMKA (KEPK–UHAMKA) with number: KEPK–NK/02/04/2025/03267. All participants signed an informed consent before participating in the study. The confidentiality of all participants was strictly maintained throughout the research process.

## 3. Results

We obtained 12 participants aged 18–22 years old who had scored 5–9 using the social media disorder scale. Table 1 shows the characteristics of the informants.

**Table 1.** Informant characteristic.

Informant code	Gender	Age
I1	Male	19
I2	Male	19
I3	Male	20
I4	Male	19
I5	Female	18
I6	Male	21
I7	Female	20
I8	Female	21
I9	Male	20
I10	Female	18
I11	Female	22
I12	Male	19

All participants have accounts on WhatsApp, LINE, Instagram, TikTok, X (formerly known as Twitter), and YouTube. Only one participant installed Facebook. Only two participants installed Telegram and Snapchat. To enhance knowledge gained from participants' individual stories, their accounts were also analyzed to enable a comprehensive exploration of their shared experiences. Nine common subthemes were identified across participants: (1) ‘Duration of social media use’, (2) ‘The second accounts of Instagram’, (3) ‘Each social media has different motivation uses’, (4) ‘Passive activity on social media’, (5) ‘Importance of peer groups’, (6) ‘Lack of openness toward parents’, (7) ‘Collective efficacy gives courage to speak up’, (8) ‘Social etiquette is a must’, and (9) ‘The shifting of cultural value during social media use’. Based on the emerging subthemes, we found four overarching themes through the SEM lens, namely “new individual digital habits”, “social network dynamics”, “community influences”, and “broader societal interplays”. These subthemes are described below.

***Overarching Theme 1: New individual digital habits***

***Subtheme 1: Duration of social media use***

The participants reported extensive daily social media use, typically exceeding three hours and ranging up to 16 hours per day, particularly during periods of boredom or inactivity. However, this usage significantly decreases during times of heightened academic or extracurricular engagement, such as college assignments, organizational commitments, or part-time work. For instance, some students acknowledged spending over eight hours a day on platforms like Instagram alone when unoccupied, often recognizing the negative impact on productivity and academic preparation. Others maintained more moderate usage, averaging six to seven hours across multiple platforms, which could decrease to two or three hours during exam periods.

*“When I'm bored, eh I can spend 8–9 hours just on Instagram, not to mention anything else, so it can be more, even 16 hours. So, it's like I'm really unhealthy (in using social media). I also feel like I have to cut back. Because I've already intended to prepare for tests, lecture materials, and others, but in the end, I don't get it done (because I'm distracted by using social media). What I haven't started gets delayed, but what's been assigned to me, I get it done, even though sometimes there's a deadline. It's like (the effort I put in) is just my bare minimum.” (I1)*

*“If the average use of Instagram and TikTok is 1–2 hours each, so all social media can total 6–7 hours per day. But if I was busy, there are many college exams, it will be reduced (in duration) to only 2–3 hours a day.” (I9)*

Notably, one participant who was juggling lectures, community practice, and part-time employment limited their social media use to just 30 minutes to an hour. These patterns highlight the situational nature of social media consumption among university students, influenced heavily by their academic and personal responsibilities.

*“(Social media usage duration) in the last 24 hours, I haven't slept, because I have a lot of activities, lectures, community practice, and part-time work, so I only use social media for 30 minutes to 1 hour.” (I12)*

***Subtheme 2: The second accounts of Instagram***

The use of multiple Instagram accounts among university students reflects a strategic approach to digital self-presentation and audience segmentation. The primary account is typically reserved for formal or curated content aimed at maintaining social image and personal branding, highlighting the importance of managing one's digital persona. In contrast, secondary and even tertiary accounts serve more informal and expressive purposes, such as sharing random daily updates, personal thoughts, or content related to hobbies like pets and food.

*“Instagram first (account) for communicating with friends, maintaining image, branding yourself. As for the second (Instagram account) for uploading random things. As for the third (Instagram account) for uploading photos of cats, food.” (I5)*

This layered usage indicates a shifting trend wherein younger users increasingly prefer the privacy and authenticity afforded by secondary accounts, which are often more active and selectively shared with closer social circles. The declining activity on primary accounts further underscores a generational shift toward more controlled and context-specific modes of online self-expression.

*“I don't know why, but nowadays (my generation) kids rarely upload on their first IG account, very rarely. While the second account is active every day, even just one story. Most of my friends are more often on the second account.” (I9)*

***Subtheme 3: Each social media has different motivation uses***

The use of various social media platforms among university students demonstrates nuanced preferences shaped by their intended communication goals, relational closeness, and platform affordances. Instagram, for instance, serves dual functions: It enables users to curate their social circle and stay updated via stories, while playing a significant role in maintaining one's image and personal branding. In contrast, Twitter is primarily used for emotional expression and informal communication with close-knit groups, often involving humor or venting. The limited number of followers on Twitter compared to Instagram reinforces its role as a more private, cathartic outlet. Moreover, TikTok emerges as a time-consuming platform for entertainment, while YouTube is preferred for acquiring comprehensive information, especially for educational purposes.

Communication for academic and institutional purposes is more structured across platforms such as LINE, Discord, WhatsApp, and Telegram. LINE is widely adopted for campus-related interactions due to its practicality, privacy (not requiring phone numbers), and features like group assignments via the “ladder” tool. Discord is appreciated for its multifunctionality, including channels, threads, and screen sharing, particularly in lecture contexts. WhatsApp is reserved for formal communication with lecturers and family, signifying a boundary between academic and personal interactions. Telegram, though less common, retains relevance for pre-university study groups that have maintained their bond. Moreover, Snapchat holds a niche appeal for its casual, ephemeral sharing culture, where features like the “fire streak” gamify continued usage. These insights underscore how platform choice reflects the social and functional priorities of university students.

*“Instagram can filter who you want to follow, you can find out news from other people from their stories.” (I11)*

*“My Twitter is only for friends with close to 20 followers, making shitposts and trying to get rid of negative emotions.” (I9)*

*“Instagram is first for communicating with friends, maintaining image, personal branding.” (I5)*

*“Line, because (activity in) universities need it. For campus, most are on LINE. But for lectures, many are on Discord too because it has many features, there are channels, threads, forums to ask questions, there is screen sharing. If Line is like WA, you only chat and send pictures.” (I6)*

*“Telegram is rarely used by friends on campus, I usually use it to chat with my study group friends during the university entrance exams, who are still close to me now. If I have close friends, I use WA, but if I chat on Line, that's not very close.” (I1)*

*“This line is for communicating with friends, group assignments using the ladder feature, college information, it's just as good because it's more private and you don't share your cellphone number.” (I9)*

*“If WA is only for groups with lecturers, for communication with faculty, and interaction with family.” (I9)*

*“If I have a lot of free time, I usually (spend my free time) on TikTok because scrolling scrolling oh it turns out it takes a long time. Whereas Instagram is more about people's lives, so sometimes I change what to do (see people's lives).” (I9)*

*“For example, to learn, keep looking for more complete information on YouTube. You can find new things on Instagram, but for more complete information, I look on YouTube.” (I11)*

*“Snapchat is like being able to post photos, but people say it's even more shit, you can upload random tissue photos. Well, on Snapchat there is something like fire, the motivation for running Snapchat is so that the fire doesn't die.” (I9)*

***Subtheme 4: Passive activity on social media***

The data reveals a shift in user behavior among university students toward more passive forms of social media engagement. Participants noted a contrast between their previous high school social media usage, characterized by active content creation and public interaction and their current preference for observing rather than participating. One participant described how, although they were once highly engaged in producing content, they now primarily consume media and only occasionally respond to friends' stories, avoiding public commenting altogether. This evolution suggests a growing tendency toward selective engagement, where social media serves more as a source of information and entertainment than a platform for expression.

*“Now, I'm more like a viewer. I feel like when I was in high school, I was very active, the time and effort I gave were like what to do, I didn't have to spend time and worry about what content to make. I rarely comment in public, for example USS feed, if my friend uploads a story, I still sometimes respond.” (I9)*

Another participant further emphasized this passive approach as a conscious strategy to reduce online conflict and maintain personal comfort. They reported a preference for direct, private communication over public interaction, noting that comments from acquaintances are infrequent and that meaningful exchanges usually occur within the context of close friendships. Even within group chats, this participant identified more as a “passive reader” than an active contributor. This passive engagement reflects a broader trend of digital self-preservation, where users prioritize emotional safety, manage social exposure, and navigate online spaces with increasing intentionality and restraint.

*“If I am more passive, one of the ways is to reduce conflict by rarely commenting, and I also prefer direct interaction. Comments from other accounts are rare, the frequency will be different if it is with close friends, usually personal comments on stories. Actually, I really enjoy being passive like this, even when I am in a group chat, I also prefer to just become a passive reader.” (I11)*

***Overarching Theme 2: Social network dynamics***

***Subtheme 5: Importance of peer groups***

The participants' narratives highlight the complex interplay between peer influence, emotional expression, and platform-specific norms in shaping social media behavior. One informant shared how initial skepticism about Twitter's emotional tone turned into participation after encouragement from peers, suggesting that platform cultures such as the acceptability of expressing anger can be adopted through social modeling. Snapchat and Twitter were mentioned as platforms where viral content and affective expression are common, illustrating how digital environments can legitimize certain behaviors. This underscores how social norms within peer groups can significantly shape not only platform adoption but also users' engagement styles, particularly in emotionally charged contexts.

*“There's Twitter and another one, Snapchat, I downloaded it because people said that news goes viral on Twitter, then my friends said that if you want to get angry on Twitter, at first huh, why get angry on Twitter, but when I tried it, oh this is what it feels like.” (I9)*

Another participant expressed a high susceptibility to peer influence, often adopting the views of those they intellectually admire, even when those views later prove flawed. This reflects a cognitive vulnerability in online spaces where persuasive opinions are abundant and critical reflection may be delayed. Moreover, emotional communication remains bounded by privacy considerations; participants noted a preference for sharing feelings through private messaging, such as WhatsApp, rather than public platforms. This privatization of emotional expression is also reinforced by generational boundaries, participants avoiding friending their parents on social media, indicating a desire to maintain autonomy and peer-oriented spaces online. Collectively, these insights suggest that social media use among young adults is deeply influenced by interpersonal dynamics and platform affordances, often balancing self-expression, social conformity, and privacy.

*“I am easily influenced by people whose brains (thinking) I approve of. But if it turns out that they are wrong, I don't realize that they are wrong, I have already been carried away by the fact that I am wrong. I have a hard time admitting to others, but once I admit it, I become a bit biased towards them. Sometimes after a long pause I realize that I am biased and should not follow their opinion.” (I1)*

*“If I share my feelings with friends, I usually use WhatsApp chat, not sharing it on public stories. Also, when it comes to using social media, friends are the ones who know better, not parents. I'm not friends with my parents on social media either haha.” (I11)*

***Subtheme 6: Lack of openness toward parents***

The participants' reflections indicate a distinct separation between academic and emotional openness, especially in their relationships with parents. One informant mentioned being generally open about college-related matters but hesitant to share personal feelings. When seeking help, the preference is to consult friends first, with parents considered only as a last resort. This prioritization suggests that peers are viewed as more accessible or emotionally safe sources of support among students, while parental involvement is reserved for situations perceived as more serious or unavoidable academic problems.

*“(Regarding) College matters, I'm open, but not really about personal feelings. Mmm (if I ask for help), I usually ask my friends first, maybe as a last resort, then my parents.” (I6)*

Another participant attributed their emotional reticence to long-standing habits developed in childhood, explaining a tendency to internalize personal problems to avoid burdening their parents. This enduring reluctance to seek parental help reflects an internalized sense of self-reliance and emotional self-containment. The concern about being a “bother” suggests not only a protective stance toward parents but also highlights potential gaps in emotional communication within family dynamics. These patterns reveal a broader trend of emotional autonomy among young adults, where social support networks are selectively activated based on the perceived nature and severity of the issue.

*“Since I was little, I always kept my problems, so if I didn't find out, (my parents) wouldn't know the problem. I rarely asked my parents for help, I thought it would be troublesome or a bother (for my parents).” (I1)*

***Overarching Theme 3: Community influences***

***Subtheme 7: Collective efficacy gives courage to speak up***

Participants noted that exposure to widespread discussions on social media, particularly regarding national socio-political issues, triggered a Fear of Missing Out (FOMO) and a desire not to appear indifferent. Although not confident in their expertise, the participant is engaged by reposting relevant content, suggesting that peer behavior and collective momentum can prompt even passive forms of participation.

*“Lately there have been some problems in Indonesia. In my faculty, students are really up-to-date with social and political issues in Indonesia. Literally all people (on Instagram) upload add yours (regarding the issue) then I became FOMO and do not want to appear as a tone-deaf.” (I7)*

Additionally, offline communities such as ethnic student associations provided emotional support and a sense of solidarity. Participant I3 described how regular bonding activities within the Bugis student association created a strong support system, making them feel encouraged and backed up. This social backing reinforced their willingness to speak up and participate in group-related concerns, underscoring the role of group cohesion in promoting self-expression.

*“The association is really going well, there are often meetings like bonding, hanging out together. (I) will feel backed up by the association of fellow Bugis” (I3)*

***Overarching Theme 4: Broader societal interplays***

***Subtheme 8: Social etiquette is a must***

The participants' responses underscore a nuanced understanding of how social norms shape new digital habits, emphasizing their role in guiding appropriate behavior rather than imposing rigid limitations on social relationships. One informant highlighted that while social norms are important, they should not dictate who one chooses to be friends with. Instead, the emphasis is placed on politeness as a foundational aspect of navigating social interactions. This perspective suggests that social norms are interpreted more as flexible guidelines for respectful behavior than as prescriptive rules that restrict personal autonomy or relational choices.

*“Social norms are important, but they don't have to limit who you're friends with. The first (most important) is more about politeness.” (I5)*

Another participant echoed a similar sentiment, framing social norms as a tool for self-regulation and emotional restraint rather than external enforcement. They described adapting their behavior such as avoiding anger and maintaining composure as a form of self-protection within social contexts. The participant also noted a willingness to adhere to social norms when they are perceived as logical, implying a selective and reflective approach to norm compliance. Collectively, these views reflect a critical stance among young adults, who acknowledge the value of social norms in maintaining civility but resist enabling them to undermine personal agency or authenticity in social relationships.

*“Well, at most, just to behave, to be more self-protective, don't get too angry, just be normal. If the social norms are logical, then I just follow them, there are no social norms that restrict me.” (I6)*

***Subtheme 9: The shifting of cultural value during social media use***

The data reveal that for most participants, cultural identity plays a minimal or nonexistent role in shaping their social media behavior. Several informants expressed uncertainty about their own ethnic background, reflecting a broader disconnection from cultural heritage in the context of digital interaction. Only one participant openly stated that cultural values were used when engaging with social media, while another admitted confusion about their mixed ethnic roots and noted that their family does not emphasize traditional cultural practices. These perspectives suggest that in everyday digital life, cultural identity may not be explicitly referenced, especially among urban or multicultural youth who engage with social media through a more individualized and generalized national identity rather than one grounded in specific ethnic traditions.

*“As for me, I never think about cultural values (playing a role) when I use social media.” (I5)*

*“I'm also confused, Sundanese but my mother is not too Sundanese, my father is Padang but not too much. Actually, he's (father) just like an average Indonesian, not too cultural. Thus, I don't know which (ethnicity) I lean more towards.” (I6)*

In contrast, one participant offered a divergent perspective, demonstrating how cultural values can significantly influence social media behavior under specific circumstances. Identifying as Bugis, an ethnic group known for its strong code of honor, the participant recounted an experience of engaging in online conflict to defend both personal and organizational integrity. For this individual, the Bugis principle of maintaining self-esteem even in the face of confrontation translates into a readiness to assert and defend personal values in digital spaces.

*“Bugis people have a principle, if our self-esteem is mocked, then we have no choice but to fight it, even to death. Influencing in social media, keep fighting it.” (I3)*

This highlights that while cultural influence on social media use may be rare among the broader group, it remains a salient factor for individuals whose identities are deeply rooted in ethnic values and who perceive social media as an extension of their sociocultural world. Subtheme 8 showed that local moral codes mattered more than ethnic tradition. Norms like *sopan santun* (politeness) shape the new digital habit, such as how college students respond to comments, what they post, and what they keep private, even if they do not label those habits as “cultural value.” These findings show that culture might have shifted and mixed with urban youth norms, class expectations, and religious values to create new ways of acting online.

## 4. Discussion

We found nine key subthemes that reflect the complex interplay between digital behavior and psychological, social, and cultural dimensions. These subthemes expand current understandings of SMD, particularly among Universitas Indonesia college students, and align with prior global studies while offering nuanced local insights. Based on the emerging subthemes, the authors found four overarching themes through the SEM perspective, namely “new individual digital habits”, “social network dynamics”, “community influences”, and “broader societal interplays”.

Grounded from the individual scope of SEM, we found an overarching theme of “new individual digital habits” that exacerbate SMD among college students. The new individual digital habits consist of duration of social media use, the second accounts of Instagram, motivation for using each social media, and passive activity on social media (subthemes 1–4). The excessive duration of social media use emerged as a prominent theme. Participants reported daily use ranging from 3 to 16 hours, especially when disengaged from academic or structured responsibilities. Studies have revealed that excessive social media use in terms of duration leads to social media disorder and mental health disorders. Ergün & Alkan (2020) aimed to evaluate predictors of exclusion in adolescents and examine the effects of SMD on exclusion, which was conducted on 684 college students in Turkey; they found that students who used social media more than three hours a day had higher SMD scores (p < 0.001) [7]. Another study in the US and Mexico revealed that college students using social media for an average of 20 hours a week reported higher rates of social media addiction [22],[23].

The layered usage of Instagram accounts reflects strategic digital identity management. Participants maintained primary accounts for curated content and image management, while secondary and tertiary accounts were used for more intimate, casual sharing. Researchers have also found that excessive use of Instagram, including managing multiple accounts, can lead to emotional fatigue and instastress (Instagram stress), mediated by addiction, as users may struggle to control their time spent on the platform, exacerbating addiction [24]. A study by Barry et al., stated that there is a correlation between the number of accounts a person has on social media with FOMO and symptoms of anxiety, symptoms of depression, and feelings of loneliness [25]. These findings highlight that problematic use may not solely stem from volume but from the emotional labor associated with managing multiple digital personas.

The motivations for using different platforms revealed a functional differentiation of social media. Instagram and TikTok were preferred for entertainment and social branding, while Twitter served as an emotional outlet, and platforms like LINE and Discord were used for academic communication. This behavior supports the Uses and Gratifications Theory, which suggests that individuals actively select media to satisfy specific needs [26]. However, the integration of academic and non-academic spaces in some platforms may increase the potential for SMD through constant connectivity and blurred boundaries between academic needs and leisure.

Participants described a shift toward passive engagement on social media. This passive behavior, often marked by content consumption rather than creation, served as a coping strategy to avoid conflict, emotional exhaustion, or overexposure. Research by Fioravanti showed that passive use of social media, particularly activities involving social comparison (e.g. monitoring others' lives), is positively associated with social media addiction. This is evidenced by findings that passive users who engage in social comparison report higher levels of Facebook addiction [27]. Passive use is often linked to problematic and prolonged social media use, which exacerbates symptoms of addiction and more severe symptomatology in adolescent girls with anorexia nervosa [28]. The transition from active posting to passive lurking may reflect self-protective tendencies but can also contribute to emotional detachment and social comparison.

College students demonstrated “social network dynamics” as the second scope of SEM triggering SMD, including peer influence and family relationships (subthemes 5–6). We found that peer groups significantly shaped both platform choice and behavioral norms. Participants adopted the tone and affordances of platforms like Twitter or Snapchat after observing peer usage, including appropriating spaces for venting or humor. This aligns with Bandura's Social Learning Theory, which highlights observational learning as a key mechanism of behavior acquisition [29]. In Nigeria, peer groups often have a significant influence on behavior, including how social media is used. College students are often introduced to social media through their peers, who encourage regular use as a way to maintain social ties and group identity [30]. Moreover, the peer group's role in legitimizing certain emotional expressions reinforces the importance of social context in the development and persistence of SMD-related behaviors.

Participants reported limited emotional openness with parents and a stronger reliance on friends for support, particularly concerning emotional or personal issues. A study conducted in Canada showed that social media addiction is associated with high conflict and low satisfaction in relationships with both mothers and fathers; adolescents with higher levels of social media addiction report more conflicts and less equality in their relationships with parents [31]. East Asians, including Southeast Asians, are highly sensitive to social contexts when judging emotional expressions, and this sensitivity is linked to the cultural goal of interdependence, consequently, emotions are often restrained [32].

The cross-level of SEM perspective shows that peer relationships emerged as the most influential aspect of their new digital habit. Social media serves as a primary space for building a sense of community, mutual support, and emotional validation instead of being open toward parents. On the other hand, a form of digital self-protection also emerged, where students choose to be passive users to maintain comfort and avoid conflict. Social relationships on social media are not only a place for sharing, but also an arena for negotiating between the need for recognition and the need for security. This pattern demonstrates how peer group norms indirectly shape new digital habits, exacerbating SMD, both in terms of how they interact and what is considered appropriate for expressing emotions.

At the community scope, students live in a digital ecosystem that encourages continuous engagement, increasing the possibility of SMD. The overarching theme of “community influences” as the third layer of SEM suggests that collective efficacy both in digital and offline settings plays a critical role in encouraging students to speak up, particularly on socio-political issues. This underscores how digital peer dynamics can mobilize political expression and activism, especially within youth populations in higher education. Moreover, offline group affiliations, such as student associations based on cultural identity, further reinforced this sense of collective support and validation. This communal atmosphere enhanced the participants' confidence to express themselves, suggesting that such associations function not only as cultural anchors but also as platforms for empowerment. These findings resonate with research indicating that social media exposure fosters social confidence through group cohesion and collective efficacy. Group cohesion, in particular, plays a more significant role in enhancing social confidence compared to group efficacy [33].

Through the lens of the SEM framework, collective efficacy encourages continuous engagement that could lead to SMD among college students. This theme demonstrates how participation in social media is often driven by the need to stay abreast of social issues. Reposting or participating in online campaigns and social issues is not always driven by personal convictions, but also by the need to appear concerned and engaged. This pattern demonstrates the emergence of a new form of individual digital habits, where social responsibility is measured by presence and participation in online social and political issues. Together, online collectivism and offline solidarity contribute to the development of students' expression, particularly in navigating complex social and political environments.

At the fourth scope of SEM, we found that “broader societal layer interplays with digital habits”. This consists of social norms and cultural values (subthemes 8–9). The social etiquette was viewed not as a constraint but as a guide for respectful digital interaction. Participants emphasized politeness and emotional restraint, noting that logical norms are willingly followed while illogical ones are dismissed. This finding reflects an adaptive engagement with social norms, where internalized civility mitigates overt conflict without compromising autonomy. Researchers have noted that fairness in rule adherence is crucial. When rules are perceived as fair, individuals are more likely to comply and exhibit less psychological resistance. This is supported by findings that fair policies with low controlling language reduce perceived threats to freedom and reactance, thereby increasing compliance [34].

Finally, cultural values were largely absent in participants' reflections on their social media use. Most did not consider their ethnic background relevant to their online behavior, and some expressed uncertainty about their cultural identity. This aligns with research indicating a growing detachment from traditional identity markers in globalized digital youth cultures. The detachment from traditional identity markers among globalized digital youth is a multifaceted process influenced by globalization, digital technologies, and changing cultural preferences [35]. This shift reflects a broader trend of identity remixing, where traditional and global elements are creatively synthesized to form new, hybrid identities [36]. A study conducted in Russia revealed that the digital transformation of society poses socio-cultural threats, including the distortion of socio-cultural identity, which underscores the ongoing relevance of ethnic and cultural identities in digital contexts [37]. Nevertheless, the one participant who cited his heritage exemplified how deeply internalized cultural scripts, such as honor and pride, can inform digital interactions under certain conditions.

The societal scope of SEM shows that most students form a new digital habit as they view social media as a neutral space not tied to local cultural values. However, on the other hand, values of politeness, empathy, and caution are also firmly held, demonstrating the existence of personal ethics in navigating the digital public sphere. The new individual digital habits prefer to project a universal and urban identity rather than an ethnic or traditional one. Only one informant demonstrated selective cultural values, particularly when related to self-esteem or group honor. This phenomenon reflects a process of cultural honor, where the younger generation attempts to adapt traditional values to a more fluid digital context. As discussed in the previous themes, some participants prioritized social etiquette in their social media use, which aligns with Indonesian cultural standards that prioritize politeness when interacting with others [38]. A study entailing Spanish students revealed the distinct associations between specific internet activities and positive youth development (PYD) dimensions; thus, it is important to promote constructive digital engagement and mitigate potentially harmful practices [39].

Overall, these findings suggest that SMD among college students is not simply about new individual digital habits, but about how they learn to coexist with technology that is almost inseparable from their daily lives. The results of this study elaborated through the lens of the Socio-Ecological Model, which places SMD within a web of interconnected influences from new individual digital habits, social network dynamics, community influences to broader societal interplays. Future interventions should consider these diverse influences, promoting digital literacy, emotional regulation, and context-sensitive approaches to mental health promotion in higher education settings.

This study is subject to several limitations that may influence the generalizability of its findings. First, we employed a qualitative design with a relatively small and homogeneous sample of 12 college students, all of whom had SMD based on the SMD Scale. While the in-depth interviews provided rich insights, the limited sample size restricts the extent to which the findings can be extrapolated to broader student populations with varying degrees of SMD severity or diverse cultural and institutional backgrounds.

Second, data collection relied entirely on online interviews, which may be affected by recall bias or social desirability bias. Participants might have underreported problematic behaviors or overstated socially acceptable justifications for their social media use. Additionally, interviews were conducted within a specific cultural context among urban Indonesian college students. Therefore, the findings may not reflect the experiences of students from rural settings or those studying in different countries with different digital norms and social expectations.

In future studies, researchers should aim to reach a wider and more varied group of students, not only those identified with SMD, but also those who may be at risk, including students from different universities, academic levels, and cultural or geographic backgrounds, such as rural areas or international contexts. This would help capture a more complete picture of how social media use impacts student life. It would also be helpful to combine methods, such as surveys, interviews, and digital activity records, to balance personal stories with more objective data. By broadening both the sample and the approach, future studies can provide deeper insights into the diverse ways students experience and manage social media in today's digital world.

## 5. Conclusions

This study provides an in-depth exploration of SMD among college students, revealing a complex interplay between excessive use, emotional needs, platform-specific norms, and peer influence. The thematic findings from duration of use to digital identity strategies and passive engagement demonstrate that SMD is not merely about time spent online, but also about the psychological functions social media serves in students' daily lives. Based on the nine emerging subthemes, we found four overarching themes through the SEM perspective, namely “new individual digital habits”, “social network dynamics”, “community influences”, and “broader societal interplays”. Ultimately, addressing SMD requires a shift from simplistic models of addiction toward a more nuanced understanding that integrates cultural context, peer dynamics, and emotional well-being. By acknowledging the socio-ecological nature of students' digital engagement and promoting adaptive coping strategies, universities, researchers, and policymakers can better support young people in navigating the challenges of the digital age.

## Use of AI tools declaration

The authors declare that we have not used Artificial Intelligence (AI) tools in the creation of this article.

## Acknowledgments (All sources of funding of the study must be disclosed)

The authors would like to thank Directorate of Research Funding and Ecosystem Universitas Indonesia (DPER UI) for the funding support through PUTI Pascasarjana/Graduate Grant Year 2024 with number: NKB–106/UN2.RST/HKP.05.00/2024.


