# Supplementary material for: Beyond a digital habit: Socio-ecological perspectives on social media disorder among Indonesian college students
Source: AIMS Public Health. 2025 Nov 27;12(4):1128–45. doi: 10.3934/publichealth.2025057 (PMC12795767; doi:10.3934/publichealth.2025057)
Supplement: Supplementary file 1 [file publichealth-12-04-057-s001.pdf]

Public Health

AIMS Public Health, 12 (4): 1128–1145.
DOI: 10.3934/publichealth.2025057
Received: 23 September 2025
Revised: 12 November 2025
Accepted: 18 November 2025
Published: 27 November 2025

https://www.aimspress.com/journal/aimsph

Research article

Beyond a digital habit: Socio-ecological perspectives on social media disorder among Indonesian college students

Ulfi Hida Zainita, Evi Martha*, Tiara Amelia and Dewi Safitri

Department of Health Education and Behavioral Sciences, Faculty of Public Health, Universitas Indonesia, Building D 1st Floor (16424), Depok, West Java, Indonesia

* **Correspondence:** Email: evie.martha@ui.ac.id; Tel: +628174883103

Supplementary 1. In-depth interview guidelines

In-depth interview guidelines for informant student with SMD

Interviewer Name : ____________________
Day & Date : ____________________
Time starts : __________
Time end : __________
Informant code : __________

Name of informant : ____________________
Age : ____________________
Religion : ____________________
Gender : Female / Male
Class Year : ____________________
Types of Undergraduate : Regular/Non-regular
Faculty : ____________________
Residence status : Boarding house / With parents
Informant's telephone : ____________________
Willing to be recorded : Yes / No

A. Social media use habits

1. Can you tell us about your activities outside of studying?

Probe:

a. Do you work part time, if yes, can you tell us about it?

b. Are you involved in an organization or community on campus or in the community?

c. Are you a member of a hobby community? If not, can you tell us what your hobbies are?

2. Can you mention what social media you have installed on your cellphone and gadgets?

Probe: For each social media platform, how many accounts do you have? Including real accounts, alternate accounts, and *fake accounts*.

3. Of the social media that you have, what is your motivation for using social media?

Probe:

a. What social media do you use most often for information seeking purposes (self-education, viewing news, and learning new things)?

b. What social media do you use most often for entertainment purposes (games, music, watching videos)?

c. What social media do you use most often for social interaction purposes (staying connected with other people)?

d. What social media do you use most often to spend your free time?

e. What social media is most often used to forget the realities of life or to vent negative emotions?

4. If you think about it, what is your main motivation for using social media? *(Probe:* seeking information, interacting, seeking entertainment, spending free time)

5. What is the total duration of time spent using all social media (*Probe:* WA, YouTube, Facebook, Instagram, TikTok, Twitter, Line) in the last 24 hours?

6. What are the benefits of social media? What are the most useful apps?

7. In your opinion, are there any negative impacts of social media? Which apps have the most negative impact?

8. In your opinion, should your habit of using social media be reduced, or is it still normal, or what?

B. Social Media Activities

1. Some sources say that social media usage is divided into two categories: active and passive. (Active: using social media for updates/reciprocal interaction. Passive: using social media only for *scrolling through timelines* without interacting with others.) Which type of user do you think you are? Can you explain why?

2. According to your habits, what do you do when you open social media? *Probe:*

a. Uploading content/status updates

b. Send messages with other people/chat

c. Commenting on other accounts' posts

d. Giving "likes" to other accounts' posts

e. Just observing other accounts like scrolling through the homepage or viewing stories

f. Listening to other people's comments on an account

3. What kind of social media use do you think makes you happier? Can you explain?

C Peer influence

1. How would you categorize best friends, close friends, casual friends, and social media friends? How do each of these friendship categories affect your life?

2. What *circle of friends do you have and how does it influence your life?*
Probe: friendship circles on campus, friendship *circles* on social media, school alumni *circles* , *friendship circles* at home, friendship *circles* at boarding houses, organizations, communities, etc.
3. In your opinion, which *circle of* friends influences you the most in real life and on social media?
4. How much do your friends' opinions on social media influence you?
Probe: Have you ever felt like you had to follow your friend's opinion?
5. How do your friends influence the decisions you make?
Probe: Either in real life or on social media
6. Have you ever used certain social media because you were influenced by friends?
Probe: For what purpose? Please explain.
7. How do friends influence the behavior of *Fear of Missing Out* on some information on social media?
Probe: Being afraid of missing out on information and being considered out of *date*
8. Have you ever used social media to the point of forgetting your obligations?
Probe: What are your obligations as a child, as a student, as a friend, as a member of an organization? Can you tell us about them?

D. Relationship with friends
1. How do you and your friends spend time together?
2. How do friends respond if you ask for help if something is bothering you?
3. How do your friends talk about and express problems between friends?
4. How do your friends respond if you do a new activity or make a new change?
Probe: support or ridicule or lecture?
5. How do your friends express their feelings and respond to your feelings if you feel angry, sad, or happy?

E. Family relationship
1. How do you and your family spend time together?
2. How does your family talk about and express problems among family members?
3. How does your family respond if you ask for help if something is bothering you?
4. How does your family respond if you do new activities or make new changes?
Probe: support or ridicule or lecture?
5. How does your family express their feelings and respond to your feelings when you feel angry, sad, or happy?

F. Collective efficacy
1. In your opinion, what is the most important self-image of yourself in the real world?
Probe: Example of self-image as a UI student who looks smart
2. What do you think is the most important self-image of yourself on social media?
Probe: Example of self-image as *a high achievement individual,* Kpop/Taylor Swift fan
3. How important do you think it is to have things in common with friends on social media/other student friends?
4. How strong do you think the bonds you have with your friends on social media/other student friends are?
5. Have you ever felt left out, felt like you didn't " *connect,"* or had trouble building bonds with your friends?

6. Do you think being part of a group influences how you see yourself?
7. Have you ever regretted or felt uncomfortable being in a certain group (social media/real world)?
8. Is there a community that encourages you to be more courageous in speaking up on social media?

G. Social norms

1. In your opinion, do you try hard to follow social norms? Can you share your experiences regarding this?
2. In your opinion, should a person always follow a set of social rules? Can you explain your reasons for this opinion?
3. In your opinion, would you be happier if you didn't try to follow societal norms?
4. In your opinion, what is your idea of a perfect world? *Probe:* Is it a world with few social expectations?
5. In your opinion, are the standards that society expects us to meet too restrictive?

H. Cultural values

1. In your opinion, what is the meaning of cultural values in your life?
2. What ethnicity do you belong to? Are ethnicity and cultural value important for you?
3. Have your ethnic or cultural values ever influenced your use of social media?
4. In your opinion, what cultural values most influence you in using social media or behaving in the real world?

Probe: Are these values more *strictly* applied in real life? If so, why? If not, why?

5. Have you ever used social media in a way that goes against your cultural values?

AIMS Press

© 2025 the Author(s), licensee AIMS Press. This is an open access article distributed under the terms of the Creative Commons Attribution License (https://creativecommons.org/licenses/by/4.0)
